# Effect of duration from lingual nerve injury to undergoing microneurosurgery on improving sensory and taste functions: retrospective study

**DOI:** 10.1186/s40902-019-0244-y

**Published:** 2019-12-27

**Authors:** Takashi Nakanishi, Yuta Yamamoto, Kensuke Tanioka, Yukari Shintani, Itaru Tojyo, Shigeyuki Fujita

**Affiliations:** 1 Department of Oral and Maxillofacial Surgery, Wakayama Medical University, 811-1 Kimiidera, Wakayama, Wakayama, 641-8509 Japan; 2 Department of Anatomy and Cell Biology, Wakayama Medical University, 811-1 Kimiidera, Wakayama, Wakayama, 641-8509 Japan; 3 Clinical Study Support Center, Wakayama Medical University, 811-1 Kimiidera, Wakayama, Wakayama, 641-8509 Japan

**Keywords:** Duration time to surgery, Lingual nerve, Microneurosurgery, Peripheral nerve recovery, Schwann cells taste function

## Abstract

**Background:**

The prognosis of recovery following microneurosurgery for injured lingual nerves varies among individual cases. This study aimed to investigate if recovery ratios of sensory and taste functions are improved by the microneurosurgery within 6 months after lingual nerve injury.

**Methods:**

We retrospectively assessed 70 patients who underwent microneurosurgery at the Wakayama Medical University Hospital for lingual nerve injuries between July 2004 and December 2016. Sensory and taste functions in lingual nerves were preoperatively evaluated using a static two-point discrimination test, an intact superficial pain/tactile sensation test, and a taste discrimination test. They were evaluated again at 12 and at 24 months postoperatively. The abundance ratio of Schwann cells in the excised traumatic neuromas was analyzed with ImageJ software following immunohistochemistry with anti S-100β antibody.

**Results:**

In early cases (microneurosurgery within 6 months after the injury), recovery ratios of sensory and taste functions were not significantly different at 24 months after microneurosurgery compared with later cases (microneurosurgery more than 6 months after the injury). Meanwhile, the ratio of patients with taste recovery within 12 months after microneurosurgery was significantly decreased in late cases compared with early cases. The abundance ratio of Schwann cells in traumatic neuroma was also significantly lower in later cases.

**Conclusion:**

Microneurosurgery more than 6 months after lingual nerve injury did not lead to decreased recovery ratio of sensory and taste functions, but it did lead to prolonged recovery of taste. This delay may be associated with a decrease in the abundance ratio of Schwann cells in traumatic neuromas.

## Background

The lingual nerves are formed by the merging of branches from the mandibular nerve with the chorda tympani, providing not only somatosensory, but also taste innervation in the mucosa of the anterior two thirds of the dorsum of the tongue [1]. They may be incidentally injured in various oral and maxillofacial surgical procedures, including lower third molar removal, submandibular gland removal, sagittal splitting of the mandibular ramus, and malignant tumor removal [2, 3]. Such lingual nerve damage reportedly occurs in between 0.21 and 23% of cases, especially during lower third molar removal [4–7]. Although most patients with lingual nerve injury have complete recovery of their sensory functions without treatment after several weeks, between approximately 0.5 and 1% of patients either do not recover or only marginally recover [8, 9]. In patients with permanent sensory disturbance, traumatic neuroma has been observed at the proximal stump of the injured lingual nerve [10]. These patients can have permanent sensory disorders, including disappearance of taste, anesthesia, and dysesthesia including allodynia. To treat such sensory and taste disorders, microneurosurgery is performed to remove the traumatic neuroma followed by suturing the proximal and distal edges of the lingual nerve [11]. The degree of functional sensory recovery (FSR) after microneurosurgery, however, varies between individuals.

Several retrospective studies have been performed to identify factors related to FSR over the past 30 years [12]. The duration from lingual nerve injury to the microneurosurgery has been associated with an improved ratio of sensory function in several studies [13–16]. Susarla et al. suggested that the ratio of FSR achievement was significantly higher in patients who underwent microneurosurgery within 90 days after lingual nerve injury than in patients who underwent microneurosurgery after that time [13]. Conversely, Robinson et al. reported no correlation between the duration from lingual nerve injury to surgery or in the distance in two-point discrimination (2PD) tests [17]. Focusing on taste recovery, the relationship between this duration and function recovery has not been widely reported specific to the lingual nerve. Thus, any associations between recoveries of sensory and taste functions and the duration between lingual nerve injury and microneurosurgery have not been elucidated.

Traumatic neuromas develop at the proximal end of an injured site and can be defined as “a non-neoplastic proliferation of Schwann cells and regenerating axons in an exaggerated response to nerve injury” or “an attempt by an injured nerve to regenerate” [18, 19]. Swaim et al. and Seddon et al. reported that the shape and location of the traumatic neuroma may be used to estimate the prognosis for peripheral nerve repair [20, 21]. Raffe classified traumatic neuromas into four types according to the forms of neuroma, noting that the type could predict the treatment outcome [22]. However, any relationship between the number of Schwann cells in traumatic neuroma and function recovery in lingual nerves have not yet been examined in retrospective studies.

This study aimed to investigate if recovery ratios of sensory and taste function are improved by the microneurosurgery within 6 months after lingual nerve injury. We also measure the abundance ratio of Schwann cells in the traumatic neuroma removed from injured lingual nerve in the microneurosurgery to investigate the relationship between the duration from lingual nerve injury to microneurosurgery and the abundance ratio of Schwann cells in traumatic neuroma.

## Methods

### Study design and patients

This was a retrospective study. We collected observation data based on the inclusion criteria; patients underwent microneurosurgery of the lingual nerve in Wakayama Medical University Hospital between July 2004 and December 2016 for lingual nerve injury caused by third molar extraction. Patients were classified into two groups: those who underwent microneurosurgery within 6 months after lingual nerve injury (early cases) and those more than 6 months after injury (later cases). This was in accordance with previous study of lingual nerve recovery [14], because some patients with lingual nerve injury can spontaneously recover the sensory and taste function within 3 months. Collection of observational data was based on opt-out consent, and collection of traumatic neuromas and normal lingual nerves was based on written informed consent. This study was performed in accordance with the Declaration of Helsinki for medical protocols and was approved by the Wakayama Medical University Institutional Review Board (Nos. 1689 and 1698).

### Evaluation of lingual nerve repair

The sensory and taste tests in the tongue were performed according to the procedures described by Fujita et al. [11]. Briefly, the criteria for achieving FSR were static 2PD < 20 mm and the presence of superficial pain/tactile sensation, light touch, and brush-stroke direction without overreaction. The criterion for functional taste recovery was improving at least one kind of taste perception compared with the results of the preoperative in taste test using Taste Disc (Sanwa Kagaku Kenkyusyo Co., Nagoya, Japan; salty: sodium chloride 1.0 mol/L, sweet: sucrose 1.0 mol/L, sour: acetic acid 0.4 mol/L, and bitter: quinine 0.1 mol/L).

### Surgical procedures

All patients were seen and evaluated by one of the authors (S F). The microneurosurgery to repair the injured lingual nerve was performed by one (S F) of the authors, when patients met the criteria for performing the microneurosurgery. The criteria were as follows: (1) no signs of recovery during close follow-up for at least 3 months (2) good in general medical condition, (3) two-point discrimination (2PD) > 20 mm in the affected area, (3) no sensation observed against cold (0 °C) or hot water (42 °C) during a temperature test in the affected area, (4) no sensation against salt, sweet, sour, or bitter observed during a taste test in the affected area, (5) no sensation against sharp touch observed during a pin-prick test in the affected area, (6) no sensation against any directions observed during a brush-stroke test in the affected area, and (7) a difference in the Semmes-Weinstein monofilament test between the affected and non-affected side. Microneurosurgery procedures for lingual nerve injury were performed in all cases as previously reported [11]. Briefly, the lingual nerve was approached by an intraoral mucosal incision and lingual flap reflection, and the scar tissues around the injured site were removed. The traumatic neuroma was excised, and direct end-to-end epineural nerve sutures without tension were performed at eight or more sites around the stump using 8-0 or 9-0 nylon.

### Immunohistochemistry in traumatic neuroma samples

The excised traumatic neuroma samples in the microneurosurgery were collected from 2013 onward and fixed in 10% neutral buffered formalin overnight. Thirty traumatic neuroma samples were embedded in paraffin and sliced longitudinally. Endogenous peroxidase activities were removed by incubation with 0.3% hydrogen peroxide for 1 h. Non-specific immunoreactivities were blocked by incubation with Blocking One (Nacalai Tesque, Inc., Kyoto, Japan) for 1 h. The sections were incubated with primary antibodies at 4 °C overnight, followed by incubation with secondary antibodies conjugated with peroxidase for 1 h. The dilution ratio of the anti-S100 beta antibody was 1/1000. Secondary antibodies were detected using 3, 3′-diaminobenzidine solution with 0.01% hydrogen peroxide, and methyl green was used for nuclear staining.

### Quantification of Schwann cells in traumatic neuroma samples

Images of the tissue sections were acquired with a microscope (Eclipse E600, Nikon, Japan), and a section from approximately the middle of each specimen was selected for analysis. To determine the abundance ratio of Schwann cells, the area of immunoreactivity against anti-S100β antibodies was measured using ImageJ software in three fields of view from the central side of the traumatic neuroma to the peripheral side. The abundance ratio of Schwann cells in each sample was calculated as the average of the immunoreacted area against anti-S100β antibodies per tissue area in three fields of view [23–25].

### Statistical analysis

The primary outcome was the ratio difference of functional sensory or taste recovery at 12 months after microneurosurgery between early and later cases. Secondary outcomes were ratio difference of allodynia appearance at 12 months after microneurosurgery between early and later cases, ratio difference of sensory or taste function recovery at 24 months after microneurosurgery between early and later cases, differences between early and later cases of ratio of functional sensory or taste recovery within 12 months after the microneurosurgery compared with those 12 to 24 months after microneurosurgery, and the abundance ratio of Schwann cells in traumatic neuroma samples between early and later cases.

All data were statistically analyzed using JMP Pro 12 (SAS Institute Inc., NC, USA). Statistical comparisons were performed between early and later cases for FSR, functional taste recovery, and the presence of allodynia using Fisher’s exact test. The distance in 2PD test and abundance ratio of Schwann cells were statistically compared between early and later cases using Student’s *t* tests. For all analyses, *P* < 0.05 was considered to be statistically significant.

## Results

**Fig. 1:**
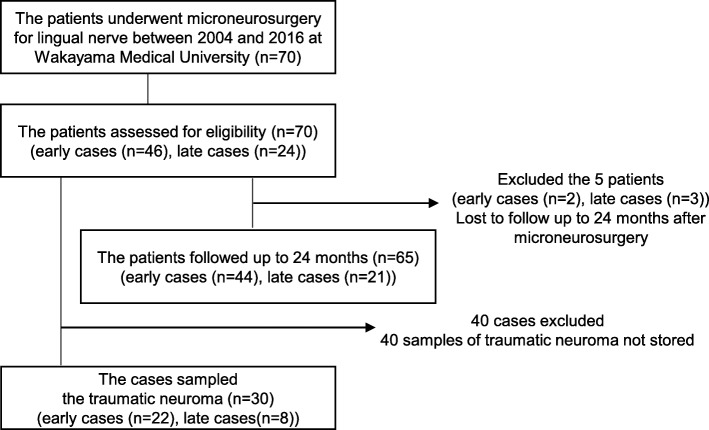
Trial profile. All patients underwent microneurosurgery for lingual nerve injury caused by third molar extraction at Wakayama Medical University Hospital between 2004 and 2016. Sixty-five out of 70 patients could be followed up to 24 months. Thirty traumatic neuroma samples were analyzed using immunohistochemistry

Seventy patients underwent microneurosurgery for lingual nerve injury caused by third molar extraction between 2004 and 2016 at Wakayama Medical University and were followed up more than 12 months after the surgery (Fig. 1). No patients were excluded according to the study eligibility criteria. Finally, 70 patients (males 19, females 51; age 36.2 ± 11.7 years) were included. Before the surgery, 36 patients had allodynia, and the average score in 2PD test was 18.7 ± 4.5. Forty-six of the 70 patients underwent microneurosurgery within 6 months after lingual nerve injury. Table 1 shows the duration between lingual nerve injury and microneurosurgery, showing age, sex, side of injury, and neuroma-in-continuity or nerve-end neuroma in each case. Thirty-six of the 70 patients had allodynia before the microneurosurgery, 20 of whom were early cases. Forty-eight of the 70 patients were > 20 mm in the 2PD test. We performed sensory and taste tests for all patients at 12 months after surgery to evaluate the relationship between the ratio of FSR achievement and the duration between injury and surgery. Five out of 70 patients were lost to follow-up and excluded from analysis of the ratio difference of sensory or taste function recovery at 24 months after microneurosurgery

**Table 1:** Patient characteristics

	Early cases	Later cases	*P* value
Sample size	46	24	–
Mean duration from injury to repair (days)	124.7 ± 41.2	725.4 ± 822.8	–
Age (years)	35.5 ± 11.9	37.4 ± 11.2	0.970
Female sex (%)	76.1	66.7	0.412
Injury on right side (%)	58.7	58.3	0.977
Neuroma-in-continuity (%)	54.4	50.0	0.804
Allodynia appearance (%)	43.4	67.0	0.081
2PD test (mm)	18.9 ± 4.3	18.4 ± 4.6	0.655

Plus–minus values are means ± SD. *P* values were calculated using Student’s *t* test or Fisher’s exact test. In the two-point discrimination test, 48 patients could not distinguish between two points 20 mm apart. The result of the two-point discrimination test in the 48 subjects was 20 mm

The 2PD tests, allodynia appearance tests, and taste discrimination tests indicated no significant difference between early and later cases at 12 months after microneurosurgery (Table 2). Furthermore, focusing on functional sensory and taste recovery, we analyzed the results of tests at 24 months after the microneurosurgery of the 65 patients who could be followed up to that point (Fig. 1). Functional sensory and taste recovery were not significantly improved in the early cases compared with later cases (*P* = 0.655, *P* = 0.586, Table 3). Meanwhile, the ratio of the patients with improving taste function in the first 12 months after the microneurosurgery compared to those in the following 12 months was remarkably higher in early cases than in later cases (*P* = 0.016, Table 4).

**Table 2:** Comparison of early and later treatment after lingual nerve injury at 12 months after surgery

	Early cases	Later cases	*P* value
2PD test (mm)	12.6 ± 4.2	11.1 ± 3.7	0.173
Allodynia appearance	6 (13.0%)	4 (16.7%)	0.857
FSR achievement	38 (82.6%)	18 (75.0%)	0.517
Recovery of taste	25 (54.4%)	9 (37.5%)	0.127

Plus–minus values are means ± SD. *P* values were calculated using Student’s *t* test or Fisher’s exact test. In the two-point discrimination test, four patients could not distinguish between two points 20 mm apart. The result of the two-point discrimination test in the four patients was 20 mm

**Table 3:** Functional sensory and taste recovery in early and later cases at 24 months after surgery

	Early cases		Later Cases		
	Improvement	No improvement	Improvement	No improvement	*P* value
FSR achievement	41 (93.2%)	3 (6.8%)	19 (90.5%)	2 (9.5%)	0.655
Recovery of taste	27 (61.4%)	17 (38.6%)	15 (71.4%)	6 (28.6%)	0.586

Numbers/percentages represent the number or ratio of cases. *P* values were calculated using the Fisher’s exact test

**Table 4:** Treatment period to improve sensory and taste function in early and later cases

	Early cases		Later cases		
	12 months	24 months	12 months	24 months	*P* value
FSR achievement	36	5	18	1	0.654
Recovery of taste	25	2	9	6	0.016

Numbers/percentages represent the number and ratio of cases. *P* values were calculated using Fisher’s exact test

**Fig. 2:**
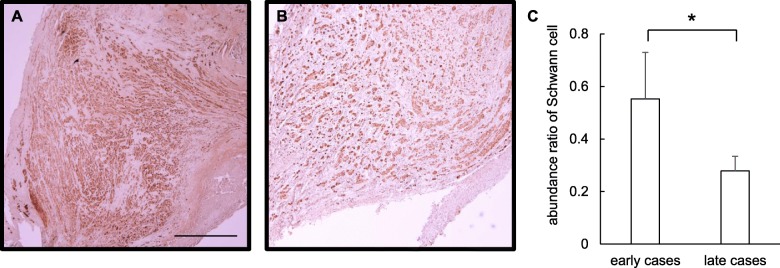
Effect of duration from lingual nerve injury to the microneurosurgery on Schwann cells in traumatic neuroma. Immunohistochemistry using anti-S100β antibody was performed on traumatic neuroma samples from early cases (**a**) and later cases (**b**). The abundance ratio of Schwann cells was significantly lower in the traumatic neuroma samples from later cases (**c**). Scale bar indicates 500 μm. Asterisk indicates significant difference between early and later cases (*P* < 0.05)

To explore the factors associated with the longer time to improve taste function in later cases than early cases, we analyzed 30 samples of traumatic neuromas fixed by formalin in the immunohistochemistry using anti-S100β antibody to recognize Schwann cells. Twenty-two of the 30 samples were from early cases, and eight were from later cases (Fig. 1). The abundance ratios of Schwann cells in traumatic neuroma were 55.3 ± 17.6% in the early cases and 27.9 ± 5.5% in the later cases (Fig. 2). The abundance ratio was significantly lower in later cases compared with early cases (*P* = 0.002).

## Discussion

In previous studies, lingual nerve recovery was associated with the duration from lingual nerve injury to microneurosurgery, but the methods of evaluation and the criteria of duration from injury to microneurosurgery varied between them (Table 5) [13–16]. Functional sensory recovery is often evaluated by 2PD and/or Semmes-Weinstein (SW) tests, and FSR achievement is defined as proper patient sensory perception, generally evaluated by 2PD and allodynia appearance. FSR may therefore better reflect patient’s sensory function than the results of a functional sensory test only. The patients undergoing microneurosurgery within 3 or 6 months after lingual nerve injury was defined as early case in previous studies (Table 5). Vincent et al. and Renton et al. indicate that some patients can spontaneously recover the sensory and taste function within 3 months [26, 27]. Thus, we perform microneurosurgery after the 3-month follow-up of the lingual nerve injury to exclude the patients who can spontaneously recover the sensory and taste function. In the current study, the patients undergoing microneurosurgery within 6 months after lingual nerve injury was defined as early case.

**Table 5:** Criteria of surgery and the evaluation methods in previous studies

Author	Criteria of period to undergo surgery after lingual nerve injury	Evaluation methods
Pogrel et al. [2]	< 3	Pogrel’s score
Susarla et al. [13]	< 3	FSR
Ziccardi et al. [15]	< 6	2PD, SW
Mozsary et al. [32]	< 6	FSR
Bagheri et al. [14]	< 6	MRCS
Robinson et al. [17]	None	2PD

In this study, there were no significant differences in the ratio of achievement of FSR between early and later cases, although the ratio of allodynia appearance before microneurosurgery tended to be lower in early cases than in later cases. Susarla et al. reported that the ratio of FSR achievement was significantly high in patients who underwent surgery within 3 months of lingual nerve injury. Bagheri et al. also reported that the odds ratio of FSR achievement was significantly high in patients who underwent surgery within 6 months of lingual nerve injury (Table 6). However, reanalysis of data used by Bagheri et al. using the chi-square test resulted in a *P* value of 0.056, and the 95% confidence interval for the odds ratio was between − 0.01 and 0.18 (Table 6). The sample size in the study of Bagheri et al. was, however, larger than that of other studies. We calculated the effect sizes of the odds ratio for FSR achievement on the duration from injury to surgery on order to adjust the sample size among the studies, and we compared the difference of odds ratio for FSR among the studies. The effect size reported by Susarla et al. was as much as double that reported by both Bagheri et al. and the current study. The surgery within 6 months after lingual nerve injury may not therefore be fully associated with FSR achievement, although surgery within 3 months of the injury may have some association.

**Table 6:** Relationship between FSR achievement and the duration from injury to the microneurosurgery in our study and in past studies

	Early cases		Later cases						
	FSR achieved	Total	FSR achieved	Total	Threshold months	*P* value, Fisher’s exact test	*P* value, chi-square test	95% CI [lower, upper]	Effect size
Current study	38	46	18	24	6	0.534	0.659	− 0.16, 0.31	0.09
Bagheri et al. [14]	125	133	76	89	6	0.037	0.056	− 0.01, 0.18	0.14
Susarla et al. [13]	13	14	31	50	3	0.047	–	0.07, 0.54	0.28

Effect sizes were calculated with Fisher’s exact test. *P* value was not calculated with chi-square test in the study by Susarla et al. because the number of patients that did not achieve FSR among the early cases was less than five

The current study did not show significant improvement of sensory or taste functions in early cases at 12 and 24 months after the microneurosurgery (Tables 2 and 3). Focusing on the patients with improving sensory and taste functions, taste function was recovered within 12 months after the microneurosurgery in almost all of the early cases. In contrast, over a year was needed for improvement of taste function in 40% of later cases whose taste function recovered (Table 4). Thus, patients who undergo microneurosurgery more than 6 months after lingual nerve injury may require more time to improved taste function compared with patients who undergo microneurosurgery within 6 months after lingual nerve injury.

Traumatic neuroma occurs at the proximal end of an injured site because of differentiation and proliferation of Schwann cells [28]. Schwann cells are critical components in nerve regeneration, including promotion of axon growth and myelination in damaged peripheral nerves [29]. Hall et al. reported that the no myelination for long time in absent axon induced apoptosis in Schwann cells, and Schwann cells were disappeared [30]. In the present study, the number of Schwann cells in late cases was significantly reduced compared with early cases based on histopathological analysis. The past reports also indicated that the number of Schwann cells was therefore decreased in the traumatic neuromas owing to the delay of the microneurosurgery [31]. Thus, the reduction of Schwann cells could suppress the nerve regeneration in sensory or taste function. The differences in the duration from injury to surgery did not affect the ratio of improvement of sensory or taste disorder but affect a period to recover taste function in this study. Therefore, the delay of the microneurosurgery after the injury may need more time to recover taste function followed by decreasing the number of Schwann cells. This histological analysis to measure the number of Schwann cells in removed traumatic neuroma may predict the period to recover taste function, and further studies should validate this hypothesis.

## Conclusion

Microneurosurgery within 6 months after lingual nerve injury was not associated with improvement of sensory and taste functions. However, a longer period was required to recover taste function in patients undergoing microneurosurgery more than 6 months after lingual nerve injury. This may be partially associated with the decrease in Schwann cells in traumatic neuromas. Clinicians should inform patients on the possible necessity of 2 years until improvement of taste function if they undergo microneurosurgery more than 6 months after lingual nerve injury.

## Data Availability

Please contact the author for data requests.

## Abbreviations

2PD: Two-point discrimination
FSR: Functional sensory recovery
SW: Semmes-Weinstein
